# Pathways through which higher neighborhood crime is longitudinally associated with greater body mass index

**DOI:** 10.1186/s12966-017-0611-y

**Published:** 2017-11-09

**Authors:** Andrea S. Richardson, Wendy M. Troxel, Madhumita Ghosh-Dastidar, Gerald P. Hunter, Robin Beckman, Natalie Colabianchi, Rebecca L. Collins, Tamara Dubowitz

**Affiliations:** 1 RAND Corporation, Health Division, 4570 Fifth Avenue, Pittsburgh, PA 15213 USA; 2 University of Michigan, School of Kinesiology, Ann Arbor, MI 48109-2013 USA

**Keywords:** Obesity, Crime, Physical activity, Neighborhood, Perceived safety, Structural equation modeling

## Abstract

**Background:**

Although crime and perceived safety are associated with obesity and body mass index (BMI), the pathways are less clear. Two likely pathways by which crime and perceived safety may impact obesity are through distress and physical activity.

**Methods:**

We examined data from 2013 to 2014 for 644 predominantly African-American adults (mean age 57 years; 77% female) living in low-income Pittsburgh, PA neighborhoods, including self-reported perceptions of safety and emotional distress, interviewer-measured height/weight, and physical activity measured via accelerometry. We used secondary data on neighborhood crime from 2011 to 2013. We built a structural equation model to examine the longitudinal direct and indirect pathways from crime to BMI through perceived safety, distress and physical activity.

**Results:**

Long-term exposure to crime was positively associated with lack of perceived safety (β = 0.11, *p* = 0.005) and lack of perceived safety was positively associated with BMI (β = 0.08, *p* = 0.03). The beneficial association between physical activity and BMI (β = −0.15, *p* < 0.001) was attenuated by a negative association between crime and physical activity (β = −0.09, *p* = 0.01). Although crime was associated with distress we found no evidence of a path from crime to BMI via distress.

**Conclusions:**

Our findings suggest decrements in perceived safety and physical activity are important processes that might explain why neighborhood crime is associated with greater BMI.

## Background

African Americans are disproportionately affected by obesity [1] and more likely to live in neighborhoods with higher crime rates than whites [2, 3]. Neighborhood crime and perceived lack of safety have been associated with psychological distress [4, 5], poor health [6–9], limited physical activity [10–14], and obesity [15, 16]. Literature on objective crime measures and their association with obesity, BMI and physical activity supports associations between neighborhood safety and activity [12, 17, 18]. However, the role of crime, varying degrees of perceived safety and the intersection of these factors in influencing resident distress, physical activity, and BMI are not well understood (see reviews [19–21]), especially in low-income, African American populations [22, 23].

Neighborhoods provide environments that may promote or limit health-related behaviors [24]. Increasing evidence has shown objectively measured crime and perceived lack of safety to increase stress, limit physical activity because of safety concerns, and therefore influence obesity [12, 25]. Several plausible pathways exist through which neighborhood crime could influence obesity. Chronic activation of the physiologic stress system resulting from crime exposure could lead to obesity, given how cortisol production increases BMI [26–29]. Another path from crime to obesity could operate through perceived lack of safety to physical activity. Understanding residents’ perceptions of neighborhood safety and their role in resident health could help identify ways to support resilience in disadvantaged neighborhoods [30]. Much of the existing evidence is based on self-reported physical activity and anthropometry, which are subject to reporting bias [31, 32]. Furthermore, much of the cross-sectional research focuses on direct associations between crime/perceived safety and obesity/BMI without considering two mediating pathways through distress and physical activity.

Among the few studies examining pathways, Roman et al., used structural (SEM) equation modeling with data from two deprived Chicago neighborhoods (*n* = 328, mean age 47 years) to examine the direct and indirect pathways from perceptions of neighborhood violence and disorder to obesity through fear of walking [5]. Perceived violence was positively associated with fear of walking but not with physical activity or obesity. However, this cross-sectional study relied on self-reported anthropometry and physical activity. A cross-sectional study of 864 adults from a low-income and ethnically mixed neighborhood in Salt Lake City, UT used objectively measured anthropometry and physical activity [11]. They found that low perceived safety was associated with high BMI and that lower moderate-to-vigorous physical activity partially explained the relationship between safety and BMI. Yet the authors were unable to examine how crime may relate to perceived safety.

It remains unclear how peoples’ perceptions of crime impact their sense of safety, in part, because no consensus exists on whether the location and timing of crime incidents influences resident perceptions. Understanding the types, location and timing of crimes and their relationship with perceptions of safety is necessary in order to robustly model an indirect pathway whereby crime influences BMI through perceived safety using the objective measure that is most strongly associated with perceived safety.

Prior studies have examined crime across a wide range of geographic areas (e.g., census block group [33], census tract [15], half-mile [15], and 1-mile [13]) surrounding residents’ homes in association with physical activity, diet and BMI. But it is unknown how proximity from crimes, or the timing at which crimes occurred, may be associated with physical activity and health. In one study, agreement was poor between objective measures of crime and perceived fear across 1-, half-, and eighth-mile buffers but agreement appeared to increase with decreasing buffer size [18]. This study was limited by small sample size (*n* = 303) and crimes were aggregated to an annual rate, ignoring more detailed timing of the occurrences (e.g., a month versus a year ago). In addition, much of the research on the role of crime in neighborhoods has not considered influences on health from crimes occurring with varying degrees of proximity [34].

In sum, limitations of research on neighborhood crime, perceptions, behaviors, obesity, and distress include the lack of studies that use objectively measured data (crime, physical activity, and BMI), to examine longitudinal pathways to resident health, and to examine patterns of association with crime across different levels of crime aggregation (over space and time). To address these gaps, this longitudinal study examined how proximity and timing of crime related to perceptions of safety in two predominantly African American low-income Pittsburgh neighborhoods. We then derived a long-term measure of neighborhood crime based on these associations. In a SEM, we tested direct and indirect pathways from long-term exposure to neighborhood crime (prior 2 years) to BMI a year later through perceived safety and the two likely additional mediating pathways, distress and physical activity.

## Methods

### Study population and participants

Pittsburgh Research on Neighborhoods, Exercise and Health (also known as ‘PHRESH Plus’) was designed to document and evaluate neighborhood investments in greenspace and housing on physical activity and active transport in lower-income African American neighborhoods in Pittsburgh. The sample includes randomly selected households from two communities who are part of a cohort followed over time. The Hill District neighborhood has and continues to undergo neighborhood economic investments, including renovation of greenspace for recreational activities. Homewood, the comparison neighborhood, although experiencing some investment and change, did not experience the same degree of investment. This study uses the PHRESH Plus baseline data (collected Spring 2013), prior to major greenspace and housing renovations, and follow-up data on the same cohort members from a sister PHRESH study (collected in Spring 2014). Data collection included neighborhood-level built and social characteristics, and detailed individual-level data. All study protocols were approved by the institution’s Institutional Review Board.

### Outcome variable: Body mass index (BMI) (2014)

Interviewers measured height (without shoes) to the nearest eighth inch using a carpenter’s square and an 8-ft folding wooden ruler marked in inches. Weight was measured to the nearest tenth of a pound using the SECA Robusta 813 digital scale. BMI was calculated as weight in kg divided by height in m^2^.

### Exposure variable: Neighborhood-level crime (2011–2013)

Incident-level crime data was provided by the City of Pittsburgh police department which contained comprehensive lists of all reported crimes in Pittsburgh for 2 years preceding the household survey administration (i.e., 2011–2013). We calculated street network distances from each household to crime locations for all types (e.g., robbery, assault, etc.) using ArcGIS 10.2. We geocoded 95% of the incidents using address information. To assess how different crime measures are associated with perceived safety we first calculated them across varying distances from the residents’ homes, summing the number of crimes that occurred within 1/10-, 1/4-, 1/2-, and 3/4-mile radial distances from each household address. To assess how timing of the criminal activity is associated with perceived safety we summed the number of crimes that occurred within 1 month, 3 months, 6 months, 1 year and 2 years prior to the date the respondent was interviewed in 2013**.** We created 20 crime measures total (e.g., crimes that occurred within last month and ½ mile of the residents’ residence) for each combination of timing and proximity. Because the crime measures are aggregated to buffers surrounding the respondents’ residence they are respondent-based exposures (i.e., a crime can be counted multiple times in the sample for different individuals depending on their residential location). Thus, we used counts of crime rather than neighborhood population-based rates.

To assess long-term exposure to neighborhood crime we used the measure that was most sensitive to perceived safety and averaged it over the 2 years preceding the interview date.

### Intermediate variables (2013)

#### Perceived safety

Study interviewers administered questionnaires and residents answered the question “Your neighborhood is safe from crime” using a five-point disagree-agree scale. We reverse coded responses so that higher values reflected lack of perceived safety because of neighborhood crime.

#### Psychological distress

The Kessler 6 (K6) psychological distress scale is a self-report instrument to assess psychological distress [35]. We chose the K6 because it is a well-validated measure of psychological distress that can detect the presence of mild to severe psychological problems with high levels of sensitivity and specificity in a wide variety of populations [36]. As such, it has indeed been linked with cortisol in prior research [37]. Data collectors asked residents the frequency with which they experienced distress symptoms (e.g., “feel hopeless”; “feel restless or fidgety”) in the last 30 days. Responses were on a five-point scale. Scores were summed into a single score where high values reflect distress. K6 scores can be interpreted as no or low distress = 0–4 points, moderate distress = 5–12 points, or severe distress = 13–24 points [38].

#### Objectively measured physical activity

Participants were given a tri-axial accelerometer (i.e., ActiGraph GT3X+) and asked to wear the device on their non-dominant wrist for 7 consecutive (24 h) days. Data were sampled at 30 hz and summarized into 60 s epochs. Non-wear was determined based on the Choi algorithm using vector magnitude values [39]. Sleep intervals were identified from daily bed and wake times reported by the participant. When these times were not available or irregular (e.g., > 12 h or < 3 h sleep interval; suspected inaccurate date recorded by the participant), further visual inspection of the raw accelerometer signal was used to supplement the recorded data. All sleep interval data was removed from analyses. Further, because participants did not report their bed times on the first day the monitor was worn, the first full 24 h of recording was removed to ensure that no sleep time was included in vector magnitude calculations. Vector magnitude is the square root of the sum of the three squared axes [e.g., (x^2^ + y^2^ + z^2^)^1/2^]. Data were processed in ActiLife v6.13.1. The daily average of vector magnitude counts per minute was averaged across all days with at least 10 h of wear time. A participant was included in the analysis if he/she had at least 3 days of valid wear and their vector magnitude counts were analyzed continuously. While no vector magnitude cut points for the ActiGraph wrist exist yet for sedentary activity, a 2000 counts per minute cut point was identified in 94 older women to be very low activity [40].

### Individual-level covariates (2013)

Below, we describe the variables we used as covariates in models. Residents provided information on date of birth, gender, education (categorized into at least some college/bachelor’s degree versus less than college), and marital/cohabitation status (married or living with a partner versus living alone). We also included annual income per capita (we imputed missing values *n* = 45). We did not control for race/ethnicity because the majority of the sample (92%) self-identified as Black or African American. To capture physical activity limitation we used their responses to the question “Does your health limit you when walking one block?” which we dichotomized to “a little or a lot” versus “not at all.” To account for unmeasured differences across neighborhoods that could confound associations between crime and obesity, we controlled for neighborhood, using a binary indicator of Hill District versus Homewood. Social networks may mitigate negative effects of crime on health and well-being [7] by providing positive support and social cohesion [41] and might also influence whether crimes occur in one’s neighborhood. Therefore, we also controlled for social network size which was measured using a previously validated scale [42]. Participants reported the number of people they knew (e.g., family, close friends, neighbors, etc.). The number of people in each category was summed to compute each participant’s social network size.

### Analytic sample

We excluded residents if they did not live in Hill District or Homewood at the time of the 2013 interview (*n* = 27) (i.e., if they had moved out of the neighborhood from initial enrollment into the study), were missing accelerometry data (*n* = 98), had less than 3 valid (≥ 10 h) accelerometer days (*n* = 15), were lost to follow up in 2014 when the BMI outcome was collected (*n* = 248), were missing crime data (*n* = 8), or were missing response to the question about perceived safety (*n* = 198). The final analytic sample included 644 adults who wore the accelerometer for an average of 5.7 ± 0.6 days. We calculated T-tests (continuous variables) and Chi-Square tests (categorical variables) to compare the included versus excluded (*n* = 397) residents. We compared all covariate, intermediate and, outcome variables between excluded individuals and individuals included, and only age and social network size differed. Those individuals excluded were younger (mean age 53 years vs 57 years among the included, *p* = < 0.001) and had fewer social network members (mean 28 vs 48 among the included, *p* = < 0.001).

## Statistical analyses

We performed descriptive analyses and tested multivariable models using Stata 14.0 (StataCorp, College Station, TX). We calculated means and standard deviations (continuous variables) and percentages (categorical variables) of individual-level – and neighborhood-level crime. To identify the timing and proximity of crime most sensitive (based on relative magnitude of association) to perceived safety, we used logit models to predict perceived safety as a function of 20 different crime measures. We estimated separate models for counts of crime by time and proximity. We controlled for age, gender, education, married or living with a partner, physical limitation, neighborhood, social network size, and household income in these crime-safety tests. To illustrate the magnitude of the associations we plotted a three-dimensional bar graph of the logit estimates (y-axis) by crime timing (x-axis) and distance (z-axis).

**Fig. 1:**
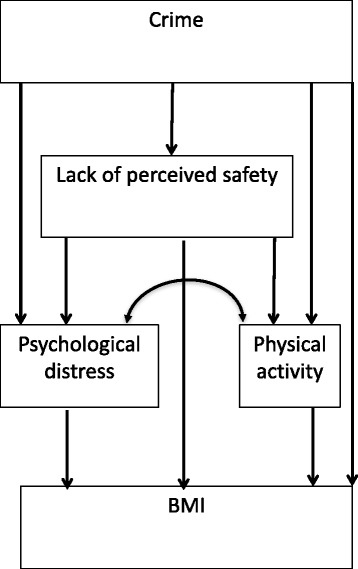
Conceptual model of direct and indirect paths from crime to BMI

To examine longitudinal indirect pathways from crime to later BMI through perceived safety, psychological distress and physical activity, we used Mplus version 7.11 [43] to build a SEM. We used the measure of crime that emerged as the best predictor of perceived safety in the above analyses and averaged it over the 2 years preceding the respondents’ interview to derive a measure that is sensitive to perceived safety and also captures long-term exposure to crime. We allowed for a direct pathway from crime to BMI. Given that there may be bidirectional associations between distress and physical activity [44, 45], we also tested whether distress and physical activity covaried (double-headed curved arrow in Fig. 1). Figure 1 presents our conceptual model. We controlled for the above-mentioned covariates to address confounding of associations between: (1) crime and BMI (exposure to outcome); (2) perceived safety, physical activity, and BMI (mediators to outcome); (3) crime, perceived safety, distress, and physical activity (exposure to mediators) that were not likely to be affected by crime [46]. A statistically non-significant Chi-Square test statistic [47], Root Mean Square Error of Approximation (RMSEA) < 0.06 [48], and Comparative Fit Index (CFI) values approaching 1.0 [49] imply the model fits the data well.

## Results

In 2013, the analytic sample was on average 57 years of age, low-income (mean per capita income $13,400), burdened with low mobility, and overweight in 2014 (mean body mass index (kg/m^2^) was 31 kg/m^2^ (Table 1 **)**.

**Table 1:** Study population characteristics in 2013 and body mass index in 2014, *n* = 644

	Mean (SD) or percentage
Body mass index (kg/m^2^)	31.1 (7.6)
Physical activity (vector magnitude^a^ [counts/min])	2139.6 (777.0)
Kessler psychological distress score (K6)^b^	4.3 (4.6)
Lack of perceived safety in neighborhood	3.8 (1.0)
Age (years)	57.0 (15.4)
Male sex	23.0%
Mean per capita annual income (in $1000)	13.5 (13.2)
Social network size	47.5 (50.4)
Education (at least some college/bachelor’s degree)	45.0%
Married or living with partner	18.9%
Any physical limitation walking 1 block	28.7%
Neighborhood	
Hill District	69.4%
Homewood	30.6%

^a^Based on 3–7 days of accelerometry data using a 1-min epoch

^b^Responses were on a five-point scale. Scores were summed into a single score (0–24) where high values reflect psychological distress

About one fifth of the cohort was married or living with a partner (19%), and an average vector magnitude of 2140 cpm suggests very low activity [40, 50]. The cohort reports relatively low distress with a mean of 4.3. The average number of crime exposures ranged from about 2 to 1424 depending on distance from residence and time frame (Table 2).

**Table 2:** Number of reported crimes (of any kind) by distance from resident’s home and time frame^a^ (2011–2013), *N* = 644

	Mean (SD)
Within last month	
Within 1/10 mile	1.9 (0.1)
Within 1/4 mile	8.8 (0.2)
Within 1/2 mile	31.0 (0.7)
Within 3/4 mile	64.2 (1.2)
Within last 3 months	
Within 1/10 mile	5.6 (0.2)
Within 1/4 mile	25.2 (0.6)
Within 1/2 mile	88.6 (2.0)
Within 3/4 mile	183.3 (3.1)
Within last 6 months	
Within 1/10 mile	10.8 (0.4)
Within 1/4 mile	47.8 (1.2)
Within 1/2 mile	167.2 (3.6)
Within 3/4 mile	346.9 (5.7)
Within last year	
Within 1/10 mile	21.4 (0.7)
Within 1/4 mile	93.6 (2.2)
Within 1/2 mile	333.5 (7.1)
Within 3/4 mile	695.5 (14.3)
Within 2 years	
Within 1/10 mile	44.4 (1.4)
Within 1/4 mile	191.9 (4.5)
Within 1/2 mile	685.2 (14.3)
Within 3/4 mile	1424.1 (21.9)

^a^Counts of crimes obtained from Pittsburgh Police Department and aggregated by timing preceding interview and radial distance from resident household

**Fig. 2:**
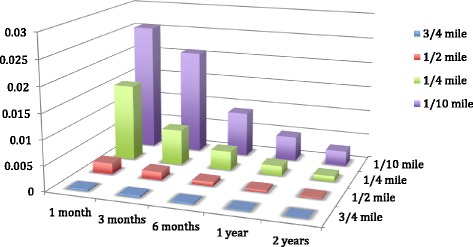
Logit coefficients from 20 models estimating lack of perceived safety as a function of crime measures that vary by time frame and distance from residents’ home

We associated 1) residents’ perceived neighborhood safety with 2) counts of crimes in varying time frames preceding the 2013 interview and 3) distance from the residence. Figure 2 shows plots of the magnitude of the logit beta estimates (y-axis) across the time frame of the crimes (x-axis) and the distance from residents’ residence (z-axis). Most estimates were statistically significant (*p* < 0.05, data not shown). Magnitude of the association between crime and perceived safety increased as distance between crime and participants’ residence decreased and as timing was closer to the interview. Perceived safety was most strongly associated with crimes that occurred within 1/10-mile from the resident’s home, and those that that happened within the month preceding the interview. Although the correlation between perceived safety and crimes within 1/10-mile and 1 month was not significant (Pearson’s correlation coefficient = 0.06, *p* = 0.11), which is likely due to the small number of crimes that occurred within 1/10 mile and the 1 month timeframe.

To address temporality, we created an average of crime over a longer period of time. First, we derived a monthly count of crime for each respondent for each month over 2 years (2011–2013) preceding the date of the resident’s interview (2013). Next, we averaged the monthly counts across the 2 years by summing the counts and dividing by 24 (mean = 1.84 crimes per month SD = 1.5). This captures the importance of crime timing to concurrent perceptions of safety, while providing an indicator of such over a longer period of time in order to better predict slow-changing BMI.

**Fig. 3:**
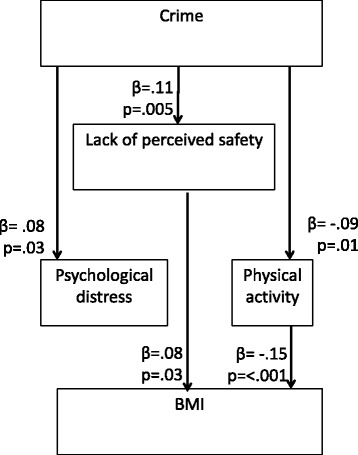
Longitudinal SEM model testing direct and indirect paths from crime to BMI, *N* = 644

In our initial model, the covariance path between distress and physical activity was not statistically significant (*p* = 0.35) and model fit was not ideal (RMSEA = 0.00, CFI = 1.00, Chi-Square = 0.00 (0 df) *p* = < 0.001). The SEM model deleting this path fit the data well (RMSEA = 0.00, CFI = 1.00, Chi-Square = 0.02 (1 df) *p* = 0.88. Figure 3 presents this result. For clarity, we present only associations that were statistically significant (*p* < 0.05). Long-term exposure to **c**rime was indirectly associated with BMI through perceived safety. We saw a positive association between the average monthly number of nearby crimes and lack of perceived safety (β = 0.11, *p* = 0.005) and lack of perceived safety was positively associated with BMI a year later (β = 0.08, *p* = 0.03). Physical activity also appeared to serve as an indirect pathway between crime and later BMI. Crime was associated with reduced physical activity (β = −0.09, *p* = 0.009), and physical activity was negatively associated with BMI (β = −0.15, *p* < 0.001). Crime was also associated with distress, but because distress was not predictive of BMI, distress does not serve as a pathway between crime and this outcome, nor does it explain associations between perceived safety and BMI. Finally, we find no direct association between crime and BMI, suggesting that the indirect paths through perceived safety and physical activity, account entirely for the association between these two variables.

## Discussion

We found that objectively measured long-term exposure to crime is associated with higher BMI at a later point in time through two separate paths**,** lack of perceived safety and decreased physical activity. Unexpectedly, distress did not explain associations between objectively measured crime, lack of perceived safety and BMI, suggesting other processes could be at play along this particular path. Future work may be warranted exploring other potential pathways. For example, lack of perceived safety may impact neighborhood cohesion or disorder and subsequently have downstream impacts on BMI via social fragmentation and isolation [51–53] that promote poor dietary behaviors. Our covariate measure of social network size may not have adequately captured such a process.

Lack of perceived safety appeared to mediate the pathway between objective crime and BMI. Thus, it is possible that perception of crime is more acutely relevant to BMI than objectively reported crime events in one’s neighborhood. This is consistent with other studies showing that perceptions of neighborhood conditions are more strongly associated with health outcomes than objective measures [54–56].

Perceived safety was not associated with physical activity, contradicting prior findings indicating that residents who felt safe in their neighborhood were more likely to walk [12, 57, 58] and be physically active [11]. However, some studies have found no association between safety perceptions and physical activity [10]. One of the first studies to examine how changes in crime and perceptions associated with walking over time found little evidence that perceived safety or police-reported crime associated with walking [13]. Fear is another reaction to crime and is a distinct construct from perceived safety, although the two constructs are correlated and both empirically linked with health [59]. Given the apparent links between neighborhood crime, fear and health [8, 60], fear may be more salient to physical activity than perceived safety. A longitudinal study in Perth, Australia, (*n* = 531) among adults (mean age 40 years) reported that perceived safety was associated with increased time walking [12]. Yet, in an earlier publication this same group reported that fear of crime was also associated with walking time and the effect size was stronger for fear than for perceived safety (22 min/wk. vs 20 min/wk). Future research should test whether fear is more salient to ones’ decision to engage in physical activity than perceived safety. While future research should examine pathways through which both perceived safety and fear may mediate associations between crime, and BMI, it is beyond the scope of this paper. Our survey asked the residents how safe their neighborhood was from crime so we opted to use this specific measure of perceived safety.

Our study population is urban, low-income, predominantly African American and sedentary so findings may not be generalizable. Given uncertainty in cut points, we analyzed vector magnitude as a continuous variable where vector magnitude is a volume measure of activity that may (or not) happen in the neighborhood. The average vector magnitude in our study was 2124.7 which is not much more than the 2000 cut point identified in 94 older women as sedentary [40]. Nonetheless, it is an important group of interest to both the study of crime and the study of overweight.

Another issue underlying inconsistencies may be the approaches used to quantify feeling safe in prior studies, such as using composite scores that include questions about walking at night and aesthetics [61, 62], unattended dogs, and safety jogging [63], or worry about being attacked [64]. These measures may tap into slightly different aspects of perceived safety or they may confound perceived safety with other variables**.** That is, composite measures of perceived safety may conflate other safety-related issues with safety specific to crime. For this reason, we opted to use a single-item measure that directly asked how safe their neighborhood was from crime.

Our finding that lack of perceived safety was associated with higher BMI is consistent with other studies [65–67]. Of the few that examine both physical activity and BMI [11, 66, 68, 69], perceived social nuisances (e.g., incivilities) among 14,836 English adults were positively associated with obesity but it was not mediated by physical activity [69]. While the strengths of Poortinga’s study include measured anthropometry and examination of mediation by physical activity they lacked objective measures of crime and physical activity. Another study among 9252 American adults living in urban areas found a positive association between county-level crime and BMI but not between county-level crime and walking [70]. Yet, this study was limited by variation within counties and by modeling physical activity and BMI separately, which does not address potential mediating pathways. Our pathway findings do support the theory that African American adults living in deprived neighborhoods are obese because crime deters physical activity [19] since we found associations between crime, perceived safety, and physical activity.

Some research suggests that people living in deprived areas with high crime rates experience stress that translates into dysregulation of the hypothalamic-pituitary-adrenal that can cause higher BMI [27, 71]. Our findings suggest that high levels of crime increase distress. In another large longitudinal study of older Australian adults that used objective measures of crime an increase in risk of experiencing distress was associated with an increase in neighborhood crime [72]. However, this study did not include perceptions of safety and our findings suggest that distress does not play a role in pathways from crime to BMI.

We also found that the association between number of crimes and lack of perceived safety got progressively stronger as the reference period was defined closer in time to interview and distance from residents’ homes was smaller. We provide evidence that when and where crimes occur is important to consider in studies that link objective measures of crime with resident perceptions of safety. Thus, researchers might not need a long reference period or wide area of assessment in studies of crime and perceived safety. However, in the case when the study outcome is slow to change, such as with BMI, it is also important to consider a method of aggregating such a measure to tap a longer history of immediate (proximal in time and space) exposure.

No consensus exists about the geographic area that best represents a neighborhood [73] and to our knowledge only one study examined how counts of crime aggregated within different distances from residents homes associated with perceived safety [18]. Among 303 adults living in Winston-Salem, NC the number of police service calls within 1-, 1/2-, and 1/8-mile and normalized by population size had low agreement with perceived safety (weighted kappas [95% CI]: 1-mile, .12 [.04–.20]; half-mile, .18 [.10–.26]; eighth-mile, .22 [.14–.30]). However, agreement appears to have been highest when using the crime rate within the closest distance. To our knowledge, no study has explored how the timing of when crimes occur influences perceptions of safety and key health outcomes.

Our study is longitudinal, capturing changes in environment, perceptions, behaviors or health. However, this study has limitations. We could not control for how long participants lived at their current address, which could contribute to a mismeasurement of their crime exposure. However, we did collect information regarding years lived in neighborhood of residence. The participants in our study are a stable population. Only 6% reported living in their neighborhood for less than a year, while 80% of the residents reported living in their neighborhood for over 5 years, and 50% of the residents reported living in their neighborhood for 20 years or more. Participants excluded from this analysis were younger which may have biased our results. Residential location choice is complex and driven by more than health-related preferences. Yet, individual behaviors and health may be tied to unobserved characteristics (e.g., health consciousness [74]) that underlie an individual’s residential location. Thus, residential selection could bias our results. We lacked dietary data at this assessment, so we were unable to test alternate pathways through energy balance. Lastly, our participants were mostly sedentary, so the lack of variation may have limited our ability to detect associations with physical activity.

Despite these limitations we present longitudinal data from a low-income and predominantly African American cohort living in underserved urban neighborhoods that includes historic crime data combined with individual-level perceptions, behaviors, and objectively measured anthropometry and physical activity. Accelerometry is superior to self-report where over-reporting can bias estimates [75]. Similarly, measured heights and weights are less vulnerable to reporting bias than self-report [32]. Further, our study population is often at increased risk of residing in disadvantaged neighborhoods [2, 3], limited physical activity [75, 76] and suffering higher rates of inactivity-related cardiometabolic conditions [77–79]. To our knowledge, this is the first analysis linking objective measures of crime and perceived safety, to psychological distress, accelerometry-derived activity, and measured anthropometry in an older, disadvantaged, and predominantly African American population.

## Conclusion

This work adds evidence that among African Americans living in urban low-income neighborhoods, lack of perceived safety in one’s neighborhood because of high crime rates is associated with higher BMI, independent of physical activity and distress. Importantly, crime is also associated with higher BMI through less physical activity, but this process is a separate one. Neighborhood investments that reduce crime and improve resident perception of safety remain critical for the wellbeing of communities. Public health professionals and policy makers may consider crime and perception of safety as salient neighborhood factors that could exacerbate obesity in the United States.

## Abbreviations

BMI: Body mass index
CFI: Comparative fit index
RMSEA: Root mean square error of approximation
SEM: Structured equation modeling
